# Graphene-assisted multiple-input high-base optical computing

**DOI:** 10.1038/srep32911

**Published:** 2016-09-08

**Authors:** Xiao Hu, Andong Wang, Mengqi Zeng, Yun Long, Long Zhu, Lei Fu, Jian Wang

**Affiliations:** 1 Wuhan National Laboratory for Optoelectronics, School of Optical and Electronic Information, Huazhong University of Science and Technology, Wuhan 430073, Hubei, China; 2 College of Chemistry and Molecular Science, Wuhan University, Wuhan 430074, Hubei, China

## Abstract

We propose graphene-assisted multiple-input high-base optical computing. We fabricate a nonlinear optical device based on a fiber pigtail cross-section coated with a single-layer graphene grown by chemical vapor deposition (CVD) method. An approach to implementing modulo 4 operations of three-input hybrid addition and subtraction of quaternary base numbers in the optical domain using multiple non-degenerate four-wave mixing (FWM) processes in graphene coated optical fiber device and (differential) quadrature phase-shift keying ((D)QPSK) signals is presented. We demonstrate 10-Gbaud modulo 4 operations of three-input quaternary hybrid addition and subtraction (A + B − C, A + C − B, B + C − A) in the experiment. The measured optical signal-to-noise ratio (OSNR) penalties for modulo 4 operations of three-input quaternary hybrid addition and subtraction (A + B − C, A + C − B, B + C − A) are measured to be less than 7 dB at a bit-error rate (BER) of 2 × 10^−3^. The BER performance as a function of the relative time offset between three signals (signal offset) is also evaluated showing favorable performance.

Graphene^1^, a conjugated sp^2^ carbon sheet arranged in a two-dimensional (2D) hexagonal lattice, has attracted a high level of research interest because of its exceptional photonic and electronic properties^2^,^3^. Graphene has linear, massless band structure E ± (p) = ±V|p|, where the upper (lower) sign corresponds to the electron (hole) band, p is the quasi-momentum, and V ≈ 10^6^ m/s is the Fermi velocity. Graphene possesses the properties of tunable Fermi level, saturable photon absorption, and adjustable refractive index^4^,^5^,^6^. Recent years have witnessed many breakthroughs in researches on graphene, including ultrafast photodetectors^7^, broadband polarizers^8^, modulators^9^, and so on. Based on the saturable absorption effect, graphene mode-locked lasers have been applied to a wide variety of laser configurations and operation wavelengths^10^,^11^,^12^,^13^. On the other hand, Hendry *et al.*^14^ have recently reported that graphene has the unique merit of possessing ultrahigh third-order nonlinear coefficient, which is corresponding to the Kerr nonlinearity, Re(χ^(3)^). It also indicates that the nonlinear response of graphene is essentially dispersionless over the wavelength. Such nonlinearity of graphene can be utilized to realize various nonlinear functional devices for telecommunications, such as optical switches, wavelength converters, and signal regenerators. Optical bistability, self-induced regenerative oscillations and four-wave mixing (FWM) have been consecutively observed in graphene-silicon hybrid optoelectronic devices^15^. FWM has also been observed in graphene in various configurations, e.g. slow-light graphene-silicon photonic crystal waveguide^16^, graphene-silicon microring resonator^17^, mechanically exfoliated graphene transferred onto fiber ferrules^18^,^19^, and graphene-based microfiber^20^,^21^. It is expected that graphene-assisted nonlinear optical devices are suitable for enabling nonlinear optical signal processing applications^18^,^19^.

Recently, spectrally efficient advanced modulation formats have been widely used in optical fiber transmission systems^22^. For optical signal processing applications, it is also attractive to employ advanced multi-level modulation formats that contain multiple constellation points in the complex plane to represent high-base numbers. Taking (differential) quadrature phase-shift keying ((D)QPSK) signal as an example, the four constellation points (i.e. four phase levels) in the complex plane can be used to represent quaternary numbers. Compared to conventional binary optical signal processing, high-base optical signal processing with advanced modulation formats could enhance the processing efficiency and capability because of the multiple bits information encoded in one symbol of a high-base number. Among various optical signal processing functions, optical computing such as addition and subtraction are basic building blocks^23^,^24^,^25^,^26^,^27^,^28^. Meanwhile, one would also expect to improve the processing throughput capacity by increasing the number of inputs (i.e. multiple-input optical signal processing)^29^,^30^. In this scenario, a laudable goal would be to combine the graphene-assisted nonlinear optical device and advanced modulation formats to enable multiple-input high-base optical computing.

In this paper, we propose an approach to performing modulo 4 operations of three-input optical addition and subtraction of quaternary base numbers using multiple non-degenerate FWM processes based on graphene coated fiber device. By adopting (D)QPSK signals (A, B, C), we demonstrate 10-Gbaud modulo 4 operations of three-input hybrid addition and subtraction of quaternary base numbers (A + B − C, A + C − B, B + C − A). The received optical signal-to-noise ratio (OSNR) penalties for modulo 4 operations of three-input quaternary hybrid addition and subtraction are measured to be less than 7 dB at bit-error rate (BER) of 2 × 10^−3^.

## Results

### Concept and working principle

**Figure 1:**
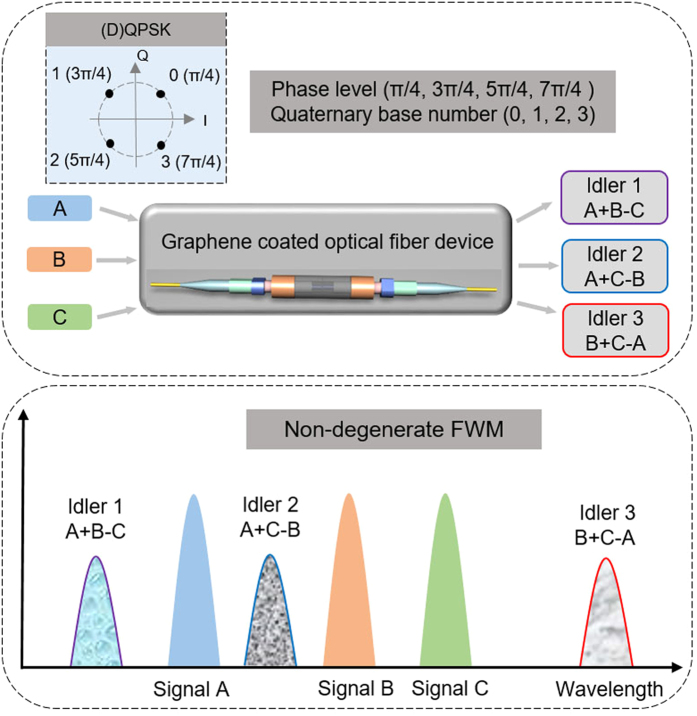
(**a**) Concept and (**b**) principle of graphene-assisted modulo 4 operations of three-input (A, B, C) quaternary hybrid addition and subtraction (A + B − C, A + C − B, B + C − A) using non-degenerate FWM and (D)QPSK signals.

Figure 1 illustrates the concept and working principle of the proposed graphene-assisted modulo 4 operations of three-input high-base optical computing, i.e. optical addition and subtraction of quaternary base numbers. From the constellations in the complex plane (i.e. I/Q plane), it is clear that we can use four-phase levels (π/4, 3π/4, 5π/4, 7π/4) of (D)QPSK to represent quaternary base numbers (0, 1, 2, 3). To implement modulo 4 operations of three-input optical quaternary addition and subtraction, a single nonlinear device (e.g. graphene coated optical fiber) is employed. Three input (D)QPSK signals (A, B, C) are launched into the nonlinear device, in which three converted idlers (idler 1, idler 2, idler 3) are simultaneously generated by three non-degenerate FWM processes. To better understand the working principle, we derive the electrical field (E) and optical phase (Ψ) relationships of three non-degenerate FWM processes under the no-depletion approximation expressed as


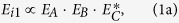



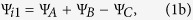



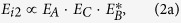



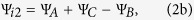



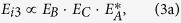



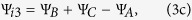


where the subscripts A, B, C, i1, i2, i3 denote input signal A, signal B, signal C, output idler 1, idler 2 and idler 3, respectively. Considering the phase wrap characteristic with a period of 2π, the linear phase relationships in Eqs (1b)(2b)(3b) imply that three converted idlers 1~3 correspond to modulo 4 operations of three-input quaternary hybrid addition and subtraction of A + B − C, A + C − B and B + C − A, respectively.

## Experimental Setup

**Figure 2:**
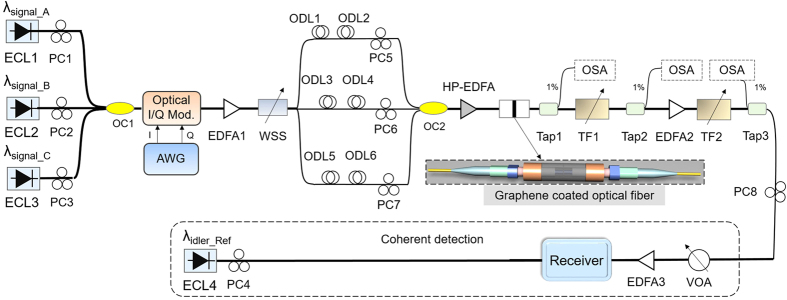
Experimental setup for graphene-assisted modulo 4 functions of three-input high-base optical computing (quaternary hybrid addition and subtraction). Inset: “sandwiched structure” graphene sample used as a nonlinear optical device. ECL: external cavity laser; PC: polarization controller; OC: optical coupler; Mod.: modulator; AWG: arbitrary waveform generator; EDFA: erbium-doped fiber amplifier; WSS: wavelength selective switch; ODL: optical delay line; HP-EDFA: high-power EDFA; TF: tunable filter; OSA: optical spectrum analyzer; VOA, variable optical attenuator.

Figure 2 shows the experimental setup for graphene-assisted three-input high-base optical computing. A single-layer graphene coated fiber device is employed. Three continuous-wave (CW) lights from three external cavity lasers (ECL1-ECL3) are sent to a (D)QPSK transmitter to produce three 10-Gbaud 2^15^ non-return-to-zero (NRZ) (D)QPSK signals (A, B, C). The three (D)QPSK signals are amplified by an erbium-doped fiber amplifier (EDFA1). A wavelength selective switch (WSS) is employed to separate three signals A, B, C. After undergoing relative integral symbols delay by tunable optical delay lines (ODLs) for decorrelation, three 10-Gbaud 2^15^ NRZ (D)QPSK signals (A, B, C) are combined together and amplified using a high-power EDFA (HP-EDFA), and then launched into the graphene coated fiber device. The polarization states of the three signals (A, B, C) are adjusted to achieve optimized conversion efficiency of non-degenerate FWM in graphene. Consequently, three converted idlers (idler 1, idler 2, idler 3) are simultaneously generated by three non-degenerate FWM processes carrying modulo 4 functions of three-input quaternary hybrid addition and subtraction of A + B − C, A + C − B and B + C − A, respectively. After the non-degenerate FWM processes, the converted idlers are selected using tunable filters (TF1, TF2), amplified by EDFA2, and then sent into the receiver for coherent detection. The CW output from ECL4 serves as a reference light for coherent detection. A variable optical attenuator (VOA) and a low noise EDFA (EDFA3) are employed to adjust the received OSNR for BER measurements. The optical spectra at different taps in the experimental setup are monitored by use of an optical spectrum analyzer (OSA) (YOKOGAWA-AQ6370C). The real-time sampling oscilloscope (Tektronix DPO72004B) operating at 50 GS/s stores the electrical waveforms for processing offline. Both ECLs at the transmitter and local oscillator (LO) laser at the receiver have a linewidth of ~100 kHz.

## Experimental Results

**Figure 3:**
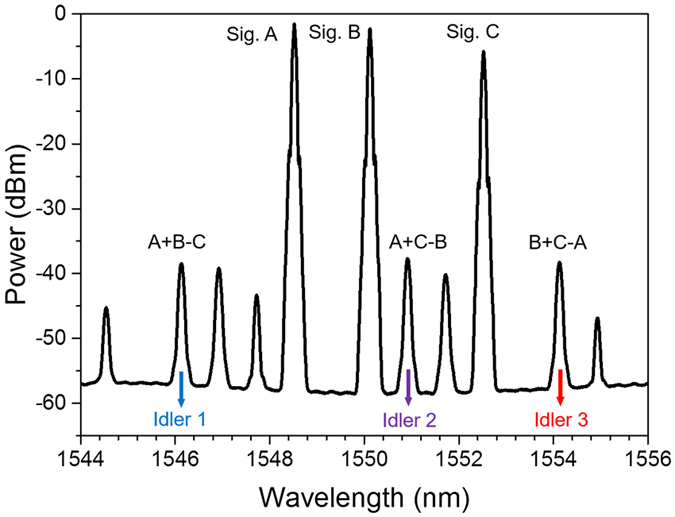
Measured spectrum for 10-Gbaud modulo 4 operations of three-input quaternary hybrid addition and subtraction (A + B − C, A + C − B, B + C − A).

**Figure 4:**
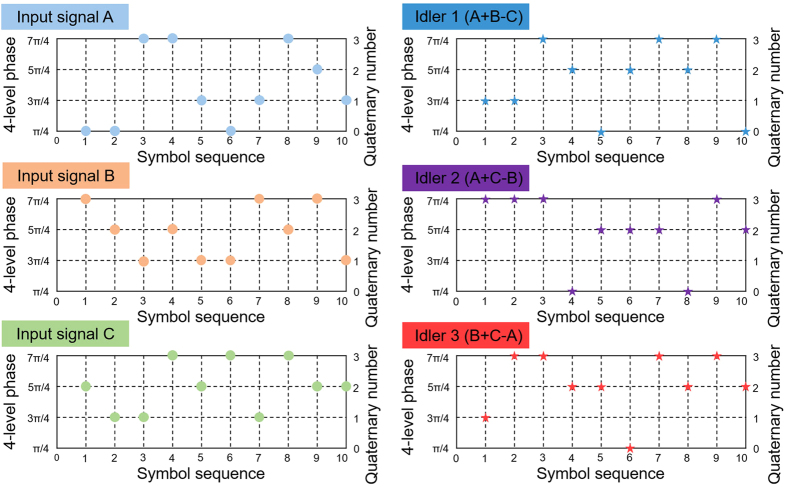
Measured phase of symbol sequence by coherent detection for 10-Gbaud modulo 4 operations of three-input quaternary hybrid addition and subtraction.

In the experiment, the wavelengths of three-input signals A, B and C are fixed at 1548.52, 1550.12 and 1552.52 nm, respectively. Figure 3 depicts measured typical optical spectrum obtained after the single-layer graphene coated fiber device. One can clearly see that three converted idlers are generated by three non-degenerate FWM processes with idler 1 at 1546.13 nm (A + B − C), idler 2 at 1550.92 nm (A + C − B), and idler 3 at 1554.13 nm (B + C − A), respectively. The power of HP-EDFA is estimated to be 31 dBm. The conversion efficiencies of three non-degenerate FWM processes are measured to be larger than −34 dB. In order to verify the quaternary optical computing functions, we measure the phase of symbol sequence for three input signals and three converted idlers, as shown in Fig. 4. By carefully comparing the quaternary base numbers for three input signals and three converted idlers, one can confirm the successful implementation of graphene-assisted modulo 4 functions of three-input quaternary optical computing (i.e. quaternary hybrid addition and subtraction) of A + B − C, A + C − B and A + C − B.

**Figure 5:**
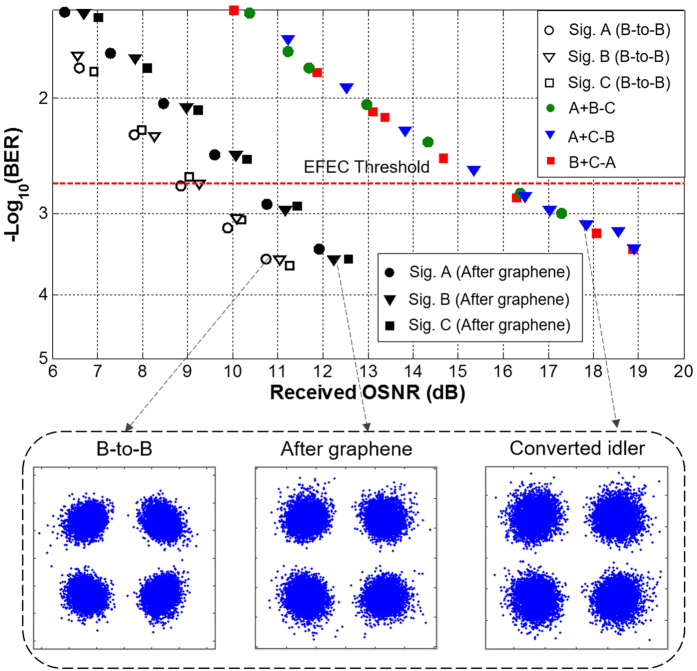
Measured BER curves for 10-Gbaud modulo 4 operations of three-input quaternary hybrid addition and subtraction of A + B − C, A − C − B, and B + C − A. Insets show constellations of (D)QPSK signals.

To characterize the performance of the proposed graphene-assisted modulo 4 functions of three-input high-base optical computing, we further measure the BER curves as a function of the received OSNR for back to back (B-to-B) signals and three converted idlers. Figure 5 depicts measured BER curves for 10-Gbaud modulo 4 operations of three-input quaternary hybrid addition and subtraction of A + B − C, A + C − B and B + C − A. As shown in Fig. 5, the observed OSNR penalties for modulo 4 operations of three-input quaternary hybrid addition and subtraction are accessed to be less than 7 dB at a BER of 2 × 10^−3^ (7% enhanced forward error correction (EFEC) threshold). The increased OSNR penalties might be mainly due to the relatively low conversion efficiency for converted idlers and accumulated distortions transferred from three-input signals (A, B, C). The insets in Fig. 5 depict corresponding constellations of the B-to-B signals and converted idlers. The BER curves and constellations of three output signals (A, B, C) after graphene are also shown in Fig. 5 for reference.

**Figure 6:**
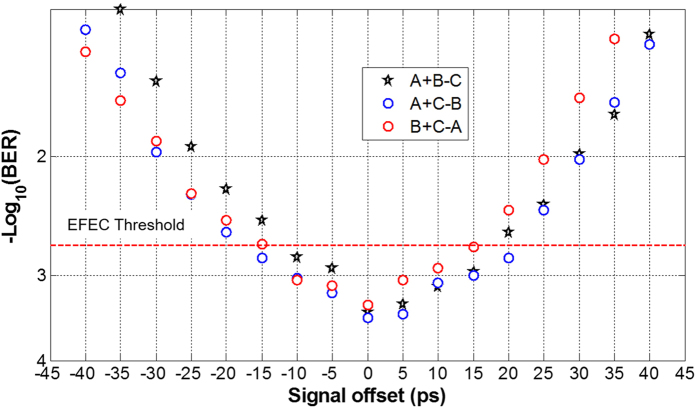
Measured BER performance versus signal offset.

For the graphene-assisted modulo 4 functions of three-input high-base optical computing, we also study the performance tolerance to the relative time offset between three input signals. Figure 6 depicts the BER performance as a function of the relative time offset between three signals (signal offset) under an OSNR of ~17 dB. It is found that the BER is kept below EFEC threshold when the signal offset is within 15 ps. The obtained results shown in Fig. 6 indicate a favorable performance tolerance to the signal offset.

## Discussion

In summary, an innovative scheme to perform graphene-assisted modulo 4 functions of three-input high-base optical computing is presented. By exploiting multiple non-degenerate FWM processes in a single-layer graphene coated fiber device and adopting (D)QPSK signals, we experimentally demonstrate 10-Gbaud modulo 4 operations of three-input quaternary hybrid addition and subtraction of A + B − C, A + C − B, and B + C − A, respectively. The received OSNR penalties at a BER of 2 × 10^−3^ are measured to be less than 7 dB for three converted idlers (A + B − C, A + C − B, B + C − A). The BER performance as a function of the relative time offset between three signals (signal offset) is also evaluated and a favorable performance tolerance is achieved. With future improvement, graphene coated fiber device might be employed to facilitate more interesting optical signal processing applications.

Remarkably, for three-input optical computing, there are, in principle, 2^3^ = 8 possibilities of the operations of A, B and C. We only demonstrate 3 of them in the experiment, i.e. A + B − C, A + C − B and B + C − A. Actually, the left 5 possibilities of the operations could be also implemented based on the same multiple non-degenerate FWM processes in graphene coated fiber device. For instance, when using –C instead of C as the input (i.e. A, B, -C), one can easily get A + B + C, A − C − B, B − C − A. As a consequence, when selectively using –A/–B/–C instead of A/B/C at the inputs, it is possible implement all the 8 possibilities of the operations of A, B and C based on the same multiple non-degenerate FWM processes in graphene coated fiber device, i.e. A + B + C, A + B − C, A − B + C, A − B − C, −A + B + C, −A + B − C, −A − B + C, −A − B − C. Note that the conversion from C to –C can be achieved using conjugated degenerate FWM process which was demonstrated in our previouis works^29^. Therefore, it is believed that all the 8 possibilities of three-input optical computing could be achieved by exploiting degenerate FWM and multiple non-degenerate FWM processes in graphene coated fiber devices.

**Figure 7:**
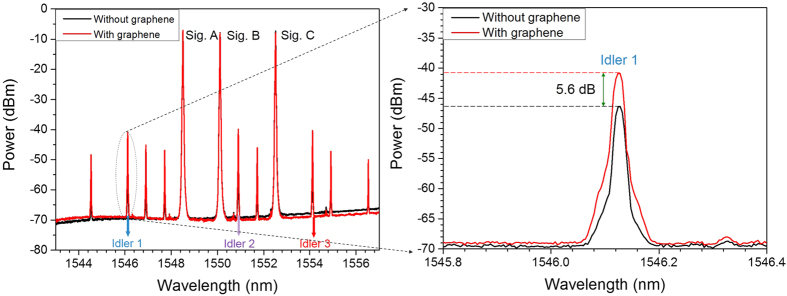
Measured spectra for multiple non-degenerate FWM processes with and without graphene coated on the end-facet of fiber. Inset: enlarged spectrum of converted idler 1.

Additionally, in order to verify the the enhancement of graphene coated fiber device, we measure the output spectrum without graphene for reference under the same experimental conditions. Moreover, we repeat the experiment by adding extra 2 m and 5 m single mode fibers in the setup and get almost the same experimental results. Figure 7 depicts the measured output spectra after the fiber with and without graphene with three input CW signals. As clearly shown in the inset of Fig. 7, taking idler 1 as an example, the power of converted idler without graphene is observed to be ~5.6 dB lower than the one with graphene. That is, under the same experimental conditions, the converted idler without graphene is ~72% lower than the one with graphene. Hence, the non-degenerate FWM processes in graphene-assisted nonlinear optical devices (e.g. graphene coated fiber device) are enhanced by comparing the conversion efficiency between the two cases with and without graphene.

## Methods

**Figure 8:**
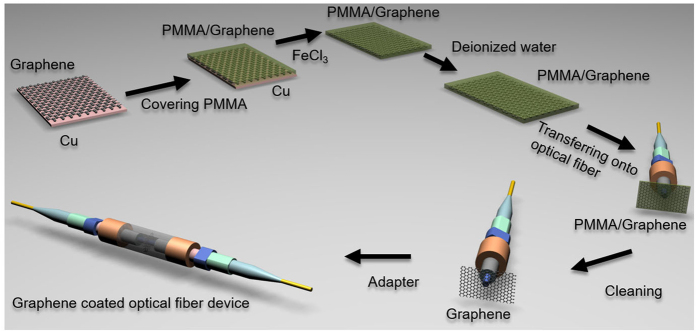
Illustration of fabrication process of the graphene-assisted nonlinear optical device.

In order to fabricate the nonlinear optical device based on a single-layer graphene, as illustrated in Fig. 8, monolayer graphene is first grown on a Cu foil (25-μm thick with a purity of >99.99 wt% obtained from Alfa Aesar) by the chemical vapor deposition (CVD) method^31^. Poly (methyl methacrylate) (PMMA) film is next spin coated on the surface of the graphene-deposited Cu foil and the Cu foil is etched away with 1 M FeCl_3_ solution. The resultant PMMA/graphene film (5 mm × 5 mm) is then washed in deionized water several times and transferred to deionized water solution or Si/SiO_2_ substrate. Then, the floating PMMA/graphene sheet is mechanically transferred onto the fiber pigtail cross-section and dried in a cabinet. After drying at room temperature for about 24 hours, the carbon atoms could be self-assembled onto the fiber end-facet thanks to the strong viscosity of graphene. The PMMA layer is finally removed by boiling acetone. By connecting this graphene-on-fiber component with another clean and dry FC/PC fiber connector, the nonlinear optical device is thereby constructed for multiple-input high-base optical computing applications based on non-degenerate FWM processes.

**Figure 9:**
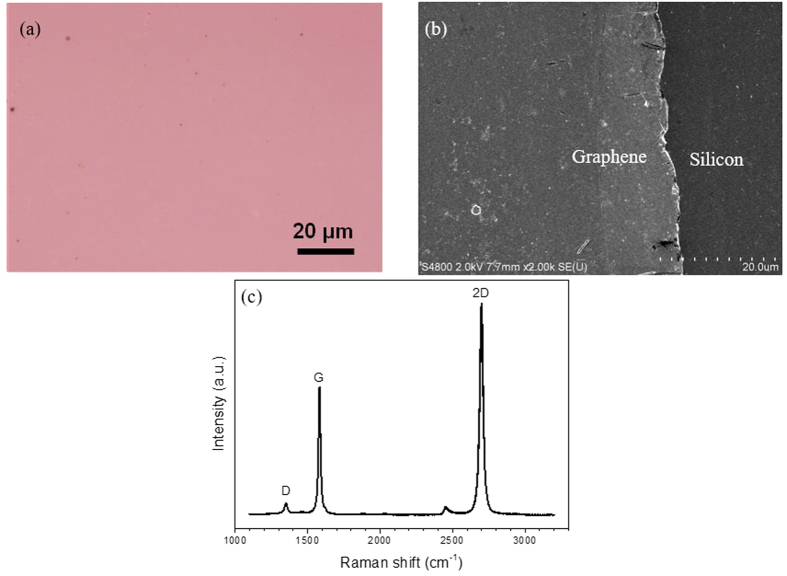
(**a**) Optical microscope image of graphene transferred on a SiO_2_/Si substrate. (**b**) Scanning electron microscope (SEM) image of graphene transferred on silicon-on-insulter (SOI). (**c**) Typical Raman spectrum of single-layer graphene on a SiO_2_/Si substrate (excitation wavelength: 532 nm).

In the experiment, optical image is taken with an optical microscope (Olympus DX51), Raman spectroscopy is performed with a laser micro-Raman spectrometer (Renishaw inVia, 532 nm excitation wavelength), and scanning electron microscopy (SEM) images are obtained by Hitachi-S4800. Figure 9(a) depicts the optical microscope image of the grown graphene film transferred on a 300 nm SiO_2_/Si substrate. The grown graphene sheet is also transferred on silicon-on-insulter (SOI) for SEM characterization, as show in Fig. 9(b). The optical microscope and SEM images shown in Fig. 9(a,b) provide evidences of the uniformity of the graphene. Selected Raman spectrum is shown in Fig. 9(c). Strong 2D and G bands are observed, accompanied by a weak D band, at 2698, 1582, and 1351 cm^−1^, respectively. The obtained I_2D_/I_G_ ratio of 1.65 demonstrates the formation of monolayer graphene^32^. The low D to G peak intensity ratios ~0.08 indicates that the graphene formed on a SiO_2_/Si substrate is almost defect-free^33^.

## Additional Information

**How to cite this article**: Hu, X. *et al.* Graphene-assisted multiple-input high-base optical computing. *Sci. Rep.* **6**, 32911; doi: 10.1038/srep32911 (2016).
